# Modeling and gene knockdown to assess the contribution of nonsense-mediated decay, premature termination, and selenocysteine insertion to the selenoprotein hierarchy

**DOI:** 10.1261/rna.055749.115

**Published:** 2016-07

**Authors:** Anze Zupanic, Catherine Meplan, Grazielle V.B. Huguenin, John E. Hesketh, Daryl P. Shanley

**Affiliations:** 1 Centre for Integrated Systems Biology of Ageing and Nutrition, Newcastle University, Newcastle-upon-Tyne NE4 5PL, United Kingdom; 2 Eawag, Institute for Aquatic Science and Technology, 8600 Dübendorf, Switzerland; 3 Institute for Cell and Molecular Biosciences and Human Nutrition Research Centre, Newcastle University, Newcastle-upon-Tyne NE2 4HH, United Kingdom; 4 Faculty of Medicine, Federal University of Rio de Janeiro, Rio de Janeiro, CEP: 21941-902, Brazil

**Keywords:** selenoprotein, mathematical modeling, translation, nonsense-mediated decay, premature termination

## Abstract

The expression of selenoproteins, a specific group of proteins that incorporates selenocysteine, is hierarchically regulated by the availability of Se, with some, but not all selenoprotein mRNA transcripts decreasing in abundance with decreasing Se. Selenocysteine insertion into the peptide chain occurs during translation following recoding of an internal UGA stop codon. There is increasing evidence that this UGA recoding competes with premature translation termination, which is followed by nonsense-mediated decay (NMD) of the transcript. In this study, we tested the hypothesis that the susceptibility of different selenoprotein mRNAs to premature termination during translation and differential sensitivity of selenoprotein transcripts to NMD are major factors in the selenoprotein hierarchy. Selenoprotein transcript abundance was measured in Caco-2 cells using real-time PCR under different Se conditions and the data obtained fitted to mathematical models of selenoprotein translation. A calibrated model that included a combination of differential sensitivity of selenoprotein transcripts to NMD and different frequency of non-NMD related premature translation termination was able to fit all the measurements. The model predictions were tested using SiRNA to knock down expression of the crucial NMD factor *UPF1* (up-frameshift protein 1) and selenoprotein mRNA expression. The calibrated model was able to predict the effect of *UPF1* knockdown on gene expression for all tested selenoproteins, except *SPS2* (selenophosphate synthetase), which itself is essential for selenoprotein synthesis. These results indicate an important role for NMD in the hierarchical regulation of selenoprotein mRNAs, with the exception of *SPS2* whose expression is likely regulated by a different mechanism.

## INTRODUCTION

The micronutrient selenium (Se) is essential for health (Rayman 2012) and its biological functions are brought about through selenoproteins, a specific group of proteins that incorporate selenocysteine, the 21st amino acid (Labunskyy et al. 2014). Selenocysteine insertion into the peptide chain occurs during translation and follows a recoding of an internal premature termination (UGA) codon (PTC) as selenocysteine, which requires a specific stem–loop structure (called the SECIS) within the 3′ untranslated region (3′UTR) of selenoprotein mRNAs (Copeland et al. 2000; Kryukov et al. 2003; Latrèche et al. 2009). It is widely assumed that recoding of the UGA codon competes with premature translation termination, which is followed by nonsense-mediated decay (NMD), a process that targets aberrant transcripts with PTCs for degradation (Berry et al. 1994; Sun et al. 2001). The expression of selenoproteins is also selectively regulated by the availability of Se, and therefore of selenocysteine, with some, but not all selenoprotein mRNAs increasing in abundance with increased availability of Se—a phenomenon that has been named the selenoprotein hierarchy (Wingler et al. 1999; Low et al. 2000; Fletcher et al. 2001). The precise mechanism(s) underlying the hierarchy is not fully understood, although it is likely that differences in the 3′UTR sequence of the different mRNAs are involved in determining competition between the mRNAs for available selenocysteine tRNA and therefore the extent to which the different mRNAs are translated.

One possibility is that different susceptibility of selenoprotein mRNAs to NMD is involved in determining the hierarchical regulation of the selenoprotein mRNAs with decreasing selenocysteine tRNA. A recent study has provided some experimental support for this hypothesis (Seyedali and Berry 2014) and earlier work showed that during Se deficiency, *GPX1* mRNA, but not *GPX4* mRNA, was targeted by NMD (Weiss and Sunde 1997; Moriarty et al. 1998). However, there is also evidence that susceptibility of NMD is not sufficient to explain the variability of selenoprotein mRNA responses to Se deficiency. A currently accepted mechanism/hypothesis proposes that only transcripts featuring a PTC of at least 50–55 nucleotides (nt) upstream of an exon junction are vulnerable to NMD (Popp and Maquat 2013). On this basis, *SELK* should not be a target of NMD, while *TXNRD2* should be. However, a recent study of dietary Se effects in rats has found that *SELK* is regulated by dietary Se, while *TXNRD2* is not (Barnes et al. 2009). Furthermore, although *GPX1*, *GPX2*, and *GPX4* are all predicted targets of NMD, their respective responses to Se deficiency are very different (Bermano et al. 1996; Wingler et al. 1999; Seyedali and Berry 2014). It has been suggested that other factors, such as differential regulation of selenoprotein expression by EIF4A3 (Budiman et al. 2009) or dependence of selenocysteine insertion on two different Sec-tRNA isoforms (Jameson and Diamond 2004), could explain the apparent resistance of *GPX4* to NMD under low Se; however, neither of these two mechanisms can explain the observed increased abundance of *GPX2* mRNA.

In this study, we used a systems biology approach to explore the mechanisms behind the hierarchical regulation of selenoprotein mRNAs. A colon adenocarcinoma cell line (Caco-2) was chosen as the experimental model system since these cells have previously been found to exhibit appropriate hierarchical regulation (Pagmantidis et al. 2005) and dietary Se levels have been implicated in colorectal cancer risk (Hughes et al. 2015). Expression of selenoprotein mRNAs in Caco-2 cells grown under different Se conditions (from Se deficiency to Se repletion) was determined experimentally and used to build mathematical models of selenoprotein translation that incorporated the different processes likely to be involved in determining the selenoprotein mRNA hierarchy. Fitting the data to the models allowed us to predict the combined effects of NMD inhibition and Se deficiency and these predictions were tested experimentally following knockdown of *UPF1*, a crucial NMD factor (Kurosaki and Maquat 2013). We show that a single model, which includes competition among selenocysteine insertion, premature termination, and NMD, and deadenylation-dependent mRNA turnover can explain the observed variations in mRNA levels for all selenoprotein mRNAs studied with the exception of *SPS2*, which is likely regulated by a different mechanism.

## RESULTS

### Effect of Se status on selenoprotein mRNA levels in Caco-2 cells

**FIGURE 1.**
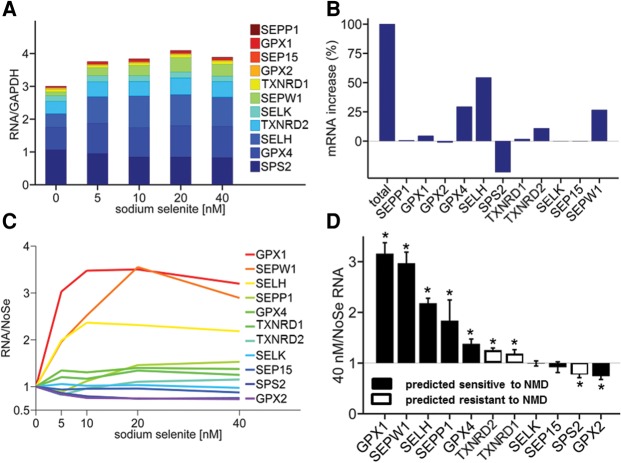
Selenoprotein mRNA abundance in Caco-2 cells grown in media supplemented with different sodium selenite concentrations. (*A*) Total selenoprotein mRNA abundance normalized to *GAPDH*. (*B*) Contribution of increase of individual selenoprotein mRNAs to the total increase in selenoprotein mRNA in high Se supplementation (40 nM) over no added Se (NoSe). (*C*) Selenoprotein mRNA abundance, normalized to mRNA abundance obtained for NoSe. (*D*) Ratio of selenoprotein mRNA abundance between high Se supplementation (40 nM) Se and NoSe. All data were produced from eight individual experiments (*n* = 8) run in duplicates. (*) *P* < 0.05, *t*-test.

To investigate the effects of Se status on selenoprotein mRNA levels in Caco-2 cells, RNA was extracted from cells grown either in media deficient in Se (NoSe) or in media supplemented with different concentrations of sodium selenite, ranging from 5 nM (considered as sub-optimal Se supply on the basis of *GPX1* expression [Crosley et al. 2007]) to 40 nM (considered as adequate Se supply [Crosley et al. 2007]). Sodium selenite was chosen as the supplement since this has been found previously to provide a better source of Se for selenoprotein synthesis in cells in culture than selenate or selenomethioinine (Hoefig et al. 2011). Se depletion had little or no effect on cell viability as judged by the absence of morphological differences, growth rate, and time to reach confluence (within 5%–10% regardless of Se status) under microscopic observations. Eleven selenoprotein mRNAs were selected for analysis based on the proposed importance of the corresponding protein in colonic function and the ability to detect their expression in Caco-2 cells under all Se conditions. Expression levels were determined using RTqPCR (Fig. 1). Compared with expression in Se-deficient conditions, a 30% increase in total selenoprotein mRNA was observed at 5 nM sodium selenite (*P* < 0.01, ANOVA), followed by a plateau (Fig. 1A). Most of this increase was due to an increase of *SELH*, *GPX4*, and *SEPW1* mRNA, while *SPS2* mRNA contribution was negative (Fig. 1B). When mRNA levels were normalized to mRNA abundance in the Se-deficient medium, Se status had a marked effect on mRNA levels for some selenoproteins but not others (Fig. 1C). A large Se-dependent increase (250%–350%) in RNA abundance was observed for *GPX1, SEPW1*, and *SELH* in cells supplemented with 5 nM sodium selenite, before reaching a plateau for added concentrations above 10 nM. A moderate increase (130%–150%) was observed for *TXNRD2* and *GPX4* mRNA levels, which reached a plateau at around 10–20 nM supplemented sodium selenite. A similar increase in RNA level was observed for *SEPP1* and *TXNRD1* mRNA but only for concentrations of sodium selenite of 20 nM and above. On the contrary, increased Se supply had little or no effect on *SELK* and *SEP15* mRNA levels. In addition, a 30% decrease in RNA levels was observed for *GPX2* and *SPS2* mRNA when the medium was supplemented with 10 nM sodium selenite or more compared with Se-deficient medium. As a result, under these conditions, *GPX1* and *SEPW1* mRNAs appeared to be the lowest in the hierarchy, being the most sensitive to reduced Se supply, *SELK* and *SEP15* were high in the hierarchy with no changes in expression associated with difference in Se supply, whereas both *GPX2* and *SPS2* responded differently in showing increased mRNA expression when Se supply was limited.

Among selenoprotein mRNAs experimentally observed as sensitive to Se supply, most were predicted to be targets of NMD, according to the 50- to 55-nt NMD rule (Fig. 1D). However, *SEP15* and *GPX2*, both predicted to be NMD targets, showed no response or a reduction in mRNA abundance, respectively, to increased Se supply. Similarly, the abundance of most selenoprotein mRNAs predicted to be resistant to NMD did not respond to changes in Se availability, except for *TXNRD1* and *TXNRD2 mRNA*, which showed a rise in response to increased Se supply in the Caco-2 cell model. There was no correlation between selenoprotein mRNA abundance and sensitivity to Se (ρ = 0.17, *P* = 0.61; Materials and Methods), indicating that mRNA abundance itself does not influence the selenoprotein hierarchy.

### Mathematical models of selenoprotein translation can fit the experimental data

**FIGURE 2.**
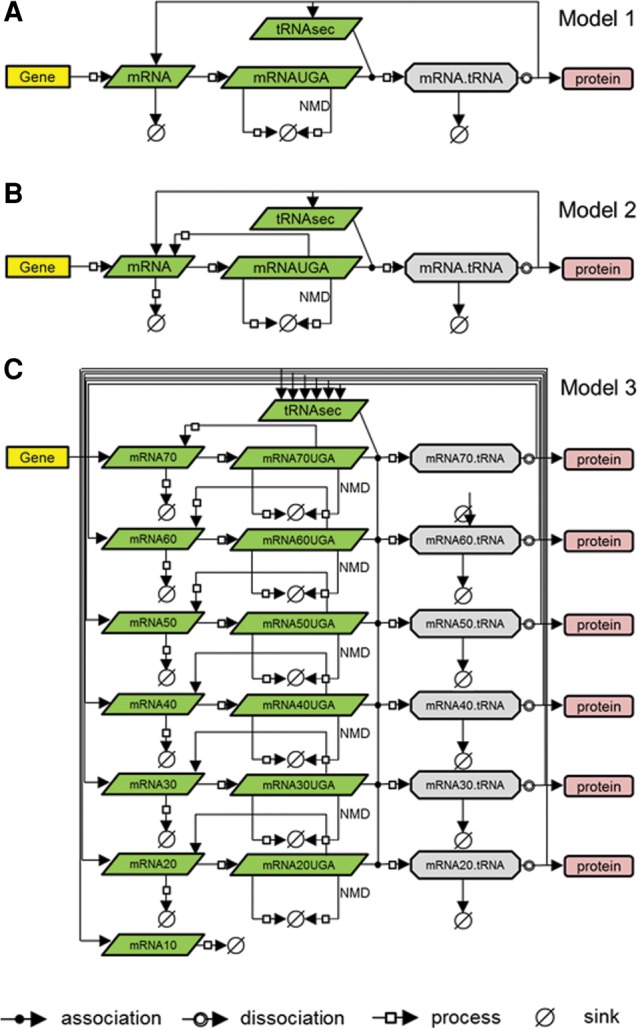
Mathematical models of selenoprotein translation in SBGN (Le Novère et al. 2009). All models include the following processes: gene transcription, ribosome binding at the initiation codon, elongation until the Sec-encoding UGA codon, binding of Sec-tRNA to the transcript, NMD of mRNA, dissociating of Sec-tRNA, elongation up to stop codon and termination of translation, degradation of the mRNA. (*A*) Model 1: competition between NMD and selenocysteine insertion. (*B*) Model 2: competition among NMD, selenocysteine insertion, and ribosome drop-off. (*C*) Model 3: competition among NMD, selenocysteine insertion, and ribosome drop-off, plus poly(A)-dependent mRNA degradation. In all models it is assumed that a constant pool of aminoacylated tRNAsec is available.

To further explore the extent to which NMD alone could explain the effects of Se status on mRNA expression of different selenoproteins, a simple process-like mathematical model of selenoprotein translation (Model 1, Fig. 2A) was built and fitted to the experimental data. The model included the following reactions: transcription, translation initiation, translation elongation until the Sec-encoding UGA codon, binding of tRNASec to the transcript, either NMD or insertion of Sec and dissociation of tRNAsec, elongation from UGA to stop codon and termination, and background degradation of the mRNA (Fig. 2A). The same model structure and initial conditions were used for modeling translation of all selenoproteins; however, the process rates were chosen according to known selenoprotein transcript features (e.g., the rate of translation from UGA to stop codon depended on the length of individual mRNAs from the UGA to the stop codon) or were used for fitting the model (Supplemental Table S1; Ingolia et al. 2011; Schwanhäusser et al. 2011; Trcek et al. 2013).

**FIGURE 3.**
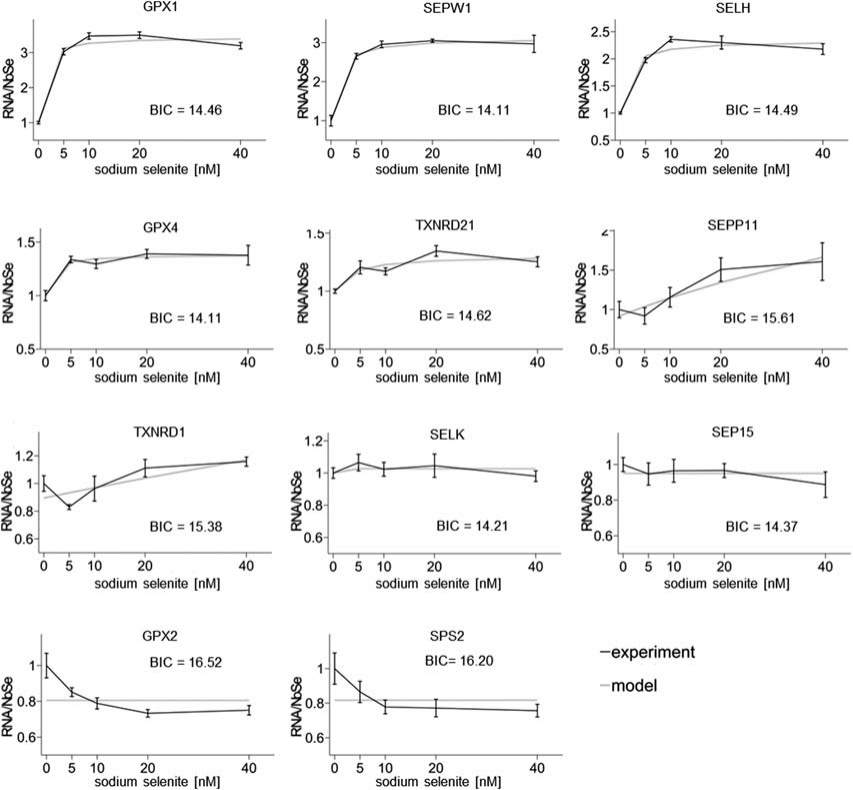
Best Model 1 fit to the experimental data for each individual selenoprotein. The quality of the model fit is given by the Bayesian information criterion (BIC) (see Materials and Methods).

Model 1 included the competition between detection of a PTC (followed by immediate NMD) and selenocysteine insertion (in essence, competition between binding of a release factor and binding of tRNAsec to the ribosome), and it provided a very good fit to the experimental mRNA abundance data for most selenoproteins; however, for *GPX2* and *SPS2*, experimental data deviated substantially from the predicted model for all data points (Fig. 3; Supplemental Table S3). In Model 1, more available Se leads to more Sec insertion and less NMD-mediated degradation, and as a result, the model predicts that only an increase in mRNA abundance is possible under such conditions. Since the experimental data show that this is not the case, the results suggest that although competition between NMD-mediated degradation and Sec-insertion can account for some of the observed effects, in particular for selenoproteins lower in the hierarchy, it cannot account for the inverse dependence of mRNA abundance on Se abundance featured by *GPX2* and *SPS2*.

Although a good fit of a model to experimental data supports its validity, there is the chance of underdetermination—when several different parameter values of a model lead to the same goodness of fit. Indeed this was the case for the nonresponsive *SELK* and *SEP15*, where Model 1 provided a good fit, but uncertainty of the inferred model parameters was high. Potentially, this uncertainty would weaken interpretation of additional modeling work on *SELK* and *SEP15* and therefore we left these transcripts out of further analysis.

As Model 1 did not fit all the experimental data, further models of selenoprotein translation were developed in which additional mechanisms that affect selenoprotein hierarchy were taken into consideration. A potential weakness of Model 1 was that it modeled each selenoprotein independently. As translation of all selenoproteins is dependent on Sec-insertion, at low Se levels selenoproteins high in the hierarchy have been proposed to compete with selenoproteins low in the hierarchy for the binding of tRNASec, causing them to undergo NMD. We modified Model 1 to incorporate aspects of competition (Model 1B, which is in essence an integration of eleven different versions of Model 1, one for each selenoprotein) (Low et al. 2000). Model 1B did not provide any improvement over Model 1 (data not shown), therefore additional models were tested.

Model 2 takes into account evidence suggesting that detection of a PTC does not always lead to NMD (Buchan and Stansfield 2007), but can also lead to normal ribosomal termination (ribosome drop-off). Therefore in this model, the competition between NMD and selenocysteine insertion is kept, but the outcome of PTC detection can either be NMD or ribosome drop-off, which leads to a free mRNA molecule (Model 2, Fig. 2B; Supplemental Table S3). However, addition of this new reaction did not improve the fit. Importantly, since Model 2 also did not include a mechanism that would stabilize the mRNA at low Se levels, we were still unable to match the experimental data for *GPX2* and *SPS2*.

**FIGURE 4.**
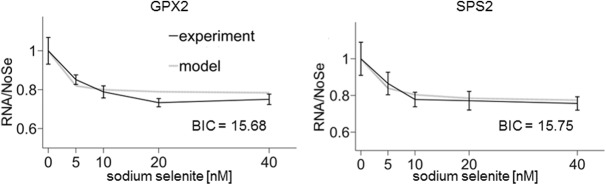
Best Model 3 fit to the experimental data for *GPX2* and *SPS2*. The quality of the model fit is given by the Bayesian information criterion (BIC).

Model 3 accounts for the fact that mRNA degradation is strongly determined by the length of the poly(A) tail of the mRNA (Model 3, Fig. 2C; Cao and Parker 2001; Funakoshi et al. 2007). In Model 3, each transcript is associated with a poly(A) tail (model adapted from Cao and Parker 2001). Each successful selenoprotein translation shortens the poly(A) (70 → 60 → … → 10; a.u.), until at a critical size mRNA degradation is triggered. If the ribosome drops off at the premature UGA, poly(A) length is maintained. While Model 3 brought no improvement of fit for most selenoproteins, it was able to fit experimental data for *SPS2* and *GPX2* better than Model 1 (Model 1/*GPX2*: BIC = 16.52; Model 1/*SPS2*: 16.20; Model 3/*GPX2*: 15.68; Model 3/*SPS2*: 15.75) (Figs. 2, 4; Supplemental Table S3). Therefore, while for the other selenoproteins the competition between NMD and selenocysteine insertion is sufficient to explain the sensitivity to Se, *SPS2* and *GPX2* seem to require the additional mechanism of ribosome drop-off and poly(A) tail length-dependent mRNA degradation which prevents the shortening of the poly(A) tail during translation and thus increases stability of the transcript. This suggests that for *SPS2* and *GPX2*, ribosome drop-off occurs more frequently than NMD, while for the other selenoproteins NMD is the more frequent occurrence.

### Model validation with NMD knockdown

Our results suggested that the selenoprotein hierarchy can be partly explained by competition among NMD, termination without NMD, and selenocysteine insertion. Therefore, changing any one of these three processes should affect each selenoprotein differently. To experimentally validate Model 3, expression of the helicase up-frameshift 1 (*UPF1*), a major component of the NMD process, was knocked down using siRNA technology. mRNAs that were either highly sensitive to changes in Se supply (*GPX1* and *SEPW1*), moderately sensitive (*GPX4*), or display a decrease in abundance when Se supply increases (*GPX2* and *SPS2*), were selected to evaluate the role of NMD on selenoprotein mRNA levels.

First, model predictions were generated for different levels of *UPF1* knockdown (KD), under the assumption that the rate of NMD decreases linearly with increased level of *UPF1* KD and that 100% *UPF1* KD should lead to an absence of the NMD process. For the five selected selenoproteins, the model predicted an increase in mRNA abundance with *UPF1* KD, with the largest increase for *GPX2* and *SPS2* without added sodium selenite at 100% KD (∼80-fold and ∼30-fold, respectively). In comparison, the predicted increase for *GPX1, GPX4*, and *SEPW1* at zero-added selenite and 100% KD was only approximately threefold. The model also predicted that the differences between different levels of *UPF1* KD for each selenoprotein would be highest for zero-added sodium selenite and would gradually disappear with higher added sodium selenite concentrations.

**FIGURE 5.**
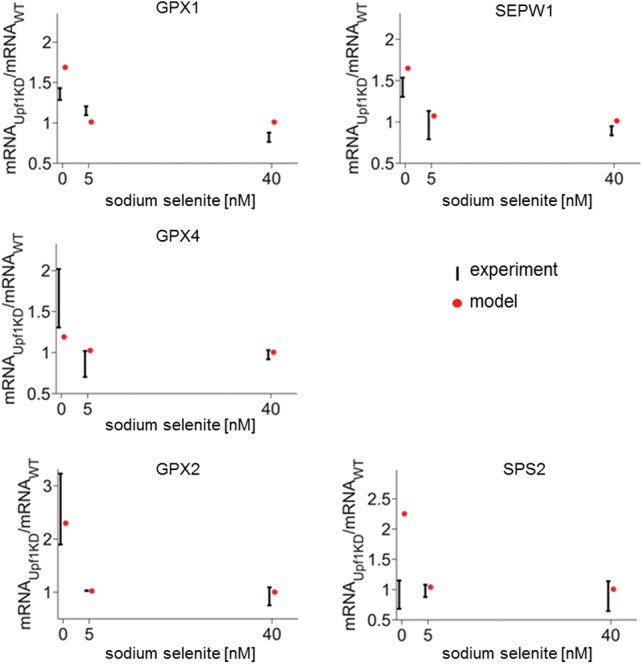
Selenoprotein mRNA abundance after *UPF1* KD. Experimental results are given in black as mean ± S.D. (*n* = 2, S.D. uncorrected), while the model predictions are given by red dots.

Model predictions were tested using RTqPCR to determine the effect of *UPF1* KD on selenoprotein mRNA abundance for different Se content. siRNA silencing was performed separately in cells grown in the absence of Se or in culture media supplemented with either 5 nM or 40 nM sodium selenite. Efficiency of *UPF1* mRNA KD was 25% after 48 h treatment with siRNA and 52% after 72 h, as assessed by quantifying *UPF1* mRNA expression compared to nonspecific KD control. The quantified selenoprotein mRNA abundances were compared with the modeling prediction at the same level of *UPF1* KD (e.g., for a KD of 25%, we imposed a reduction of 25% of the NMD rate in the model). The effects of *UPF1* KD on *GPX1*, *GPX2*, *GPX4*, and *SEPW1* are as predicted by the model (Fig. 5). In contrast, the model predictions did not match the experimentally observed *SPS2* mRNA abundances, which were found not to be sensitive to *UPF1* KD (Fig. 5). This indicates that Model 3 offers a plausible explanation for the observed selenoprotein mRNA hierarchy for most selenoproteins, but not for *SPS2*, which requires a different mechanism to explain the sensitivity of its mRNA expression to Se.

## DISCUSSION

Although the phenomenon of the hierarchical regulation of selenoprotein mRNA expression under limited Se availability is well documented (Wingler et al. 1999; Hesketh 2008), the mechanisms behind it are not yet fully understood (Sunde and Raines 2011). The results of the present study are for most selenoprotein mRNAs compatible with a competition among three processes that can occur when the ribosome reaches the internal UGA codon: selenocysteine insertion, premature translation termination, and NMD. The exception was *SPS2*, which is regulated by another mechanism. While we do not provide an explanation for the special status of *SPS2* among the selenoproteins, its role in the synthesis of selenocysteine and thus in regulation of its own protein synthesis and the synthesis of all the other selenoproteins might be the underlying cause.

Recent evidence, combined with earlier studies, strongly indicates that competition between NMD and selenocysteine insertion plays a crucial role in regulation of selenoprotein expression: (i) Either by in vitro transcription experiments (Toyoda et al. 1990; Bermano et al. 1995; Wingler et al. 1999) or by measuring both nuclear and cytoplasmic transcripts abundance (Moriarty et al. 1998), it has been shown that regulation of selenoprotein expression by Se is neither due to transcriptional regulations nor to mRNA processing, but occurs at the level of selenoprotein translation and/or mRNA stability; (ii) predicted susceptibility of transcripts to NMD, according to the currently accepted models of mammalian NMD, correlates with sensitivity to Se deficiency (Seyedali and Berry 2014); (iii) knocking down *SMG1,* a factor that was shown to be required for NMD, eliminates selenoprotein mRNA sensitivity to Se deficiency (Seyedali and Berry 2014); and (iv) increasing Se content increases the stability of selenoprotein mRNAs (Seyedali and Berry 2014). Our experimental results support and are consistent with those of Seyedali and Berry. Although they used a different cell model (HEK293T), the sensitivities of different selenoproteins to Se status in both studies are compatible, with the exception of *GPX4,* which was found to be insensitive to changes in Se supply and to NMD in HEK293T cells but to be moderately sensitive to both in Caco2 cells. This could reflect a difference in the selenoprotein hierarchy between cells of kidney and colon origin. Previously, *GPX4* has been found to be moderately sensitive to Se supply in several other cell types including liver (Bermano et al. 1995). An alternative explanation for the discrepancy is that the known *GPX4* isoforms could be differentially susceptible to NMD, and that not the same isoform dominates in different cell types. In the present study, the primer for *GPX4* recognized all *GPX4* isoforms. For *SPS2,* both studies concluded that NMD cannot explain its sensitivity to Se status.

The present study provides both computational and experimental models that support a role of NMD in the control of hierarchical regulation of selenoprotein mRNAs and are consistent with the hypothesis that competition between NMD and selenocysteine insertion plays a crucial role in selenoprotein expression regulation. Another combined experimental and computational study before ours was unable to offer a model explanation (NMD competition with selenocysteine insertion) of the increase in *GPX2* abundance under low Se conditions (Wingler et al. 1999). We show here that by including normal termination as the third competing process, *GPX2* measurements can be explained by our computational model. By using the same model to predict the effects of NMD inhibition on the mRNA abundances of different selenoproteins and experimentally verifying the predictions, we provide additional validation of the model for all tested selenoproteins, except *SPS2* (Fig. 5).

Indirect support for the presented model (competition between NMD, Sec insertion and normal termination) also derives from recent ribosome profiling studies. If there is substantial normal termination at the UGA codon, one would expect that the ribosome density (obtained by ribosome profiling, a measure of the presence of translating ribosome on transcripts) for selenoproteins would be higher before the UGA codon and lower after it. This was observed in two recent studies (Ingolia et al. 2011; Howard et al. 2013). While it is possible that the observed change in ribosome density could be the result of NMD, recent measurements show that NMD in the cytoplasm is an inherently quick process that probably would not be detected with ribosome profiling (Trcek et al. 2013). Additionally, in our recent study where we looked for changes in ribosome profiles across the genome, NMD targets were not identified (Zupanic et al. 2014).

It is important to stress that in our work we have only tested the suitability of the NMD hypotheses to explain the hierarchical regulation of selenoprotein mRNAs and showed that it explains all experimental measurements (except for *SPS2*), but we did not test any of the competing explanations of the hierarchy. Another proposed mechanism is the use of two different tRNAsec isoforms in selenocysteine insertion by selenoprotein mRNAs and a change in expression of one of the isoforms when Se supply is low (Howard et al. 2007, 2013). Although an effect of the tRNAsec isoforms has been demonstrated, it was rather small, and it is not clear how it could be used to explain the rather large change in mRNA abundance observed at low Se concentrations. Another possible mechanism is the recently discovered regulation of selenoprotein by miRNAs: mi-185 has been shown to have an effect on *GPX2* and *SPS2*. Since NMD cannot explain the experimental observations for *SPS2*, it is possible that miRNA regulation is the dominant mechanism for this selenoprotein (Maciel-Dominguez et al. 2013).

Our study did not take into account the specific molecular mechanisms behind the hierarchy. In the future, the interactions between *cis*-regulatory features, such as the SECIS, sequence surrounding the UGA and SREs, and selenoprotein translation factors, such as SBP2, EEFSEC, NCL, SECP43, EIF4A3, and tRNAsec (Low et al. 2000; Small-Howard et al. 2006; Squires et al. 2007; Budiman et al. 2009; Fixsen and Howard 2010; Caban and Copeland 2012; Gonzalez-Flores et al. 2012), should be included in the model and independently tested. Further work is required to relate our model of how NMD is related not only to the hierarchical regulation of selenoprotein mRNAs but also to the higher level of expression of the encoded proteins.

Our model suggests that NMD is inefficient for *GPX2*, while the positions of the stop codon with respect to the intron suggests otherwise. Thus, the results suggest that selenoprotein mRNAs do not play by the canonical rules of what constitutes a good target for NMD. We propose two possible solutions: either selenoprotein mRNAs in general do not conform to the rules of NMD or that the competition with selenocysteine insertion (not present for other NMD targets) and normal termination (which could very well be affected by the selenocysteine insertion machinery) render it apparently less efficient.

In conclusion, our results show that a single mechanism, namely competition among the selenocysteine insertion, NMD, and normal termination, can explain the hierarchical regulation of selenoprotein mRNAs, with the exception of *SPS2*. Experiments that would measure the length of the poly(A) chain of the selenoproteins during with and without NMD KD would be able to provide definite proof (Cao and Parker 2003). It is interesting to speculate that the competition among selenocysteine insertion, NMD, and normal termination could also be responsible for the different hierarchies of selenoproteins in different tissues, e.g., *GPX4* mRNA was shown to increase with Se in liver, but decrease in muscle. It has been shown that Se levels differ between tissues (Schomburg and Schweizer 2009) and so does NMD (Zetoune et al. 2008); however, a systematic comparison of both has not yet been performed in a single organism.

## MATERIALS AND METHODS

### Cell culture and treatment

Human colon adenocarcinoma cells (Caco-2) were grown at 37°C in a 5% CO_2_ atmosphere in Dulbecco's modified essential medium with 4.5 g/L glucose and Glutamax (Invitrogen) supplemented with 10% fetal calf serum (Sigma), 1% (v/v) penicillin–streptomycin (Invitrogen), and 1% nonessential amino acid (Invitrogen). For depletion supplementation experiments, cells were transferred 1 d after being passaged to a serum-free DMEM medium containing 0.1% (v/v) penicillin–streptomycin, 1% (v/v) (100 units/mL), insulin (5 µg/mL), and transferrin (5 µg/mL) without (Se-deficient medium) or supplemented with sodium selenite to provide an equivalent of 5–40 nM Se-repleted medium. After 3 d culture in Se-deficient or Se-supplemented medium, as used previously (Pagmantidis et al. 2005), cells were harvested and total RNA extracted. Microscopic observation was used to assess the effects of various Se levels on cell viability.

### siRNA-mediated down-regulation of *UPF1*

Knockdown of expression of *UPF1* was achieved by transient transfection of Caco-2 cells with a pool of four distinct siRNA (SI03120432, SI02629963, SI00045605, SI00045598) targeting *UPF1* (QIAGEN) or with AllStars Negative Control siRNA (QIAGEN) in the presence of Hi-Perfect transfection reagent according to manufacturer's protocol. After 48–72 h, cells were harvested and RNA extracted.

### RNA extraction and reverse transcription

Total RNA was extracted from Caco-2 cells as described in Pagmantidis et al. (2008). Briefly, culture medium was removed, cells were washed twice in ice-cold PBS, harvested and homogenized in 1 mL TRIzol reagent (Ambion, Paisley), and then frozen overnight at −80°C. After thawing on ice, TRIzol was removed by adding 0.2 mL chloroform/mL of TRIzol and samples centrifuged for 15 min, 12 000*g*, 4°C. To increase the purity of the RNA, this was followed by an extraction in 0.4 mL of phenol:chloroform:isoamyl alcohol (25:24:1)/mL of TRIzol and centrifugation for 15 min, 12,000*g*, 4°C. Phenol was removed by addition of 0.2 mL chloroform, gentle vortexing and centrifugation for 15 min, 12,000*g*, 4°C. Finally, RNA was precipitated by incubating the upper phase in 0.5 mL isopropanol per mL TRIzol for 30 min on ice followed by centrifugation for 30 min, 12,000*g*, 4°C. The pellet was washed in 75% DNase/RNase-free EtOH, dried for 5 min in vacuum desiccator and finally resuspended in 40 µL of DNase/RNase free water and quantified by nanodrop and RNA quality assessed by the *A*_260_/*A*_280_ ratio.

Reverse transcription of 0.5 µg total RNA was carried out using Transcriptor Reverse Transcriptase kit (Roche) in the presence of 100 pmol oligo(dT), according to the manufacturer's protocol. Reverse transcripts were frozen at −20°C immediately after synthesis.

### Real-time quantitative PCR

The qPCR reactions were performed in duplicate using 2 µL of 50-fold (or fivefold for *UPF1* siRNA knockdown) diluted cDNA in 20 µL reaction mixtures using SYBR Green Master mix (QIAGEN) used as a fluorescent reporter, and selenoprotein specific primers (250 nM for each primer; see Supplemental Table S2). The qPCR reactions included an initial denaturation (95°C, 5 min), followed by 45 cycles of amplification (denaturation: 95°C, 10 sec; touchdown annealing from 55°C to 60°C depending on primer annealing temperature, 15 sec; elongation: 72°C, 5 sec), and a melt curve analysis (from 65°C to 95°C) followed by a rapid cooling step at 40°C for 30 sec. The specificities of the PCR amplifications were assessed by examination of the melt curves to confirm the presence of single gene-specific peaks. Standard curves (five standards in duplicate) were generated for each amplicon from serial dilution of purified PCR products corresponding to the same sequence amplified using a RotorGene Q platform. Absolute quantification of RNA was achieved by comparing the fluorescence of samples with the amplification of the standards and normalized to *GAPDH*.

### Mathematical modeling and statistics

Mathematical models of selenoprotein translation (see Results) were built and simulated in PottersWheel (Maiwald and Timmer 2008) and are available as Supplemental Material. The model reactions and their rates for fixed parameters and physiological ranges for the free parameters were chosen from the literature and can be found in Supplemental Table S1. In particular, transcription and RNA degradation rates were taken from the global gene expression data set in mouse from Schwanhäusser et al. (2011), translation rates were taken from a ribosome profiling study in mice of Ingolia et al. (2011), and NMD-related rates were taken from measurements of the NMD rates in Trcek et al. (2013). The free model parameters were fitted to the experimental measurements of selenoprotein mRNA abundance in PottersWheel, iterating between the trust region algorithm and the genetic algorithm. The fitting was run at least a hundred times for each model/experimental data combination. The quality of the fit was evaluated using the Bayesian information criterion (BIC). BIC is based on the likelihood function and includes penalization of the number of model parameters (if two models fit the data equally well, the model with fewer parameters is preferred).

We tested the robustness of the model fitting using identifiability analysis. Briefly, identifiability analysis tells whether there is a single set of model parameters that best described the data or many different parameter sets that produce the same quality of fit. In this case, the obtained parameter values need to be taken with caution and further validation is required. We ran the identifiability analysis for each model/experimental data combination in PottersWheel using the profile likelihood method, as described by Raue et al. (2009).

Correlation between ranked mRNA abundance and rank in the selenoprotein hierarchy was evaluated using the Spearman's rank correlation coefficient. The abundance ranking was based on mRNA abundance at 10 nM supplemented sodium selenite, while the selenoprotein hierarchy ranking was based on the ratio of mRNA abundance between 40 nM and 0 nM sodium selenite supplementation.

## SUPPLEMENTAL MATERIAL

Supplemental material is available for this article.

## Supplementary Material

Supplemental Material
