# Supplementary material for: Modeling and gene knockdown to assess the contribution of nonsense-mediated decay, premature termination, and selenocysteine insertion to the selenoprotein hierarchy
Source: RNA. 2016 Jul;22(7):1076–84. doi: 10.1261/rna.055749.115 (PMC4911915; doi:10.1261/rna.055749.115)
Supplement: Supplemental Material [file supp_22_7_1076__index.html]

Modeling and gene knockdown to assess the contribution of nonsense-mediated decay, premature termination, and selenocysteine insertion to the selenoprotein hierarchy — Modeling and gene knockdown to assess the contribution of nonsense-mediated decay, premature termination, and selenocysteine insertion to the selenoprotein hierarchy — Supplemental Material 

# Modeling and gene knockdown to assess the contribution of nonsense-mediated decay, premature termination, and selenocysteine insertion to the selenoprotein hierarchy

## Supplemental Material

- Supplemental\_Tables.pdf
- Supp\_models.txt
