# Supplementary material for: Modeling and gene knockdown to assess the contribution of nonsense-mediated decay, premature termination, and selenocysteine insertion to the selenoprotein hierarchy
Source: RNA. 2016 Jul;22(7):1076–84. doi: 10.1261/rna.055749.115 (PMC4911915; doi:10.1261/rna.055749.115)
Supplement: Supplemental Material [file supp_055749.115_Supplemental_Tables.pdf]

Table S1. Model reactions and reaction rates.

Reaction Name	Description	Reaction	Parameter	Type	Value/range	Unit	Reference
Transcription	Transcription as a single step	Gene -> mRNA	k_T	free	[1e-5, 1]	1/s	(1)
Translation_1	Translation up to UGA codon	mRNA -> mRNA.UGA	k_1	fixed	25/(#codons to UGA)	1/s	(2)
tRNAsec Binding	Binding of tRNAsec to mRNA	mRNA.UGA + tRNAsec -> mRNA.sec	k_2	free	[1e-7,1]	ml/(mmol*s)	
Translation_2	Translation from UGA codon to termination	mRNA.sec -> tRNAsec + mRNA	k_{sec}	fixed	25/(#codons from UGA)	1/s	(2)
NMD	Premature termination at UGA codon, followed by nonsense-mediated decay	mRNA.UGA ->	k_{NMD}	free	[1e-7,10]	1/s	(1,3)
Background Degradation 1	Degradation of mRNA with ribosome at UGA	mRNA.UGA ->	k_D	fixed	2.5e-4	1/s	(1)
Background Degradation 2	Degradation of free mRNA	mRNA ->	k_D	fixed	2.5e-4	1/s	(1)
Background Degradation 3	Degradation of mRNA with ribosome after UGA	mRNA.sec ->	k_D	fixed	2.5e-4	1/s	(1)
Ribosome drop-off	Normal termination at premature UGA codon	mRNA.UGA->mRNA	k_{DROP}	free	[2.5e-4,1e2]	1/s	(1,3)

References

1. Schwanhausser, B., Busse, D., Li, N., Dittmar, G., Schuchhardt, J., Wolf, J., Chen, W. and Selbach, M. (2011) Global quantification of mammalian gene expression control. *Nature*, **473**, 337-342.
2. Ingolia, N., Lareau, L. and Weissman, J. (2011) Ribosome profiling of mouse embryonic stem cells reveals the complexity and dynamics of mammalian proteomes. *Cell*, **147**, 789-802.
3. Trcek, T., Sato, H., Singer, R. and Maquat, L. (2013) Temporal and spatial characterization of nonsense-mediated mRNA decay. *Genes & development*, **27**, 541-551.

Table S2. Primers for rtPCR.

**Gene**	**primer**	**sequence**	**Annealing Temperature (˚C)**
GPX1	hGPX1RT-Fwd	5'-CAAGAACGAAGAGATTCTGAATT-3'	60
	hGPX1RT-Rev	5'-CACTTCTCGAAGAGCATGAA-3'	
GPX2	hGPx2RT-Fwd	5'-GACATCAGGAGAACTGTCAGAA-3'	60
	hGPX2RT-Rev	5'-CATTTTTGGACAAGGGTGAAG-3'	
GPX4	hGPX4RT-Fwd	5'-CGATACGCTGAGTGTGGTTTGC-3'	60
	hGPX4RT-Rev	5'-CATTTCCCAGGATGCCCTTG-3'	
SELH	hSelHRT-Fwd	5'-TTGTTATCGAGCATTGCACTAGC-3'	60
	hSelHRT-Rev	5'-AACTCTTCCACCACCTCTTGAG-3	
SELK	hSelKRT-Fwd	5'-TGGAAGAGGGCCACCAGGAA-3'	60
	hSelKRT-Rev	5'-ATGCGCATGTCCGGTTGTCT-3'	
SEP15	hSeP15RT-Fwd	5'-CTTTGCAGCTCTTGTGATCT-3'	57
	hSeP15RT-Rev	5'-CATACAGCTTTTTGGTTTCAAA-3'	
SEPP1	SePP1RT-Fwd	5'-TGCCTTTTTCCTTCCTAACTT-3'	55
	SePP1RT-Rev	5'-CTTCATCTTTGAGAGTCGTGA-3'	
SEPW1	hSePW1RT-Fwd	5'-AAGTCCAAGTATCTTCAGCTCAAG-3'	60
	hSePW1RT-Rev	5'-TCTTCTTAGAGTGAATCAACTTC-3'	
SPS2	hSPS2RT-Fwd	5'-GTCCTTTGTTATTCATAATCTGCC-3'	60
	hSPS2RT-Rev	5'-GTTTCAGCTGAGGTTCCTTG-3'	
TXNRD1	hTR1RT-Fwd	5'-GACAGTTCGTACCAATTAAAGTTGAAC -3'	60
	hTR1RT-Rev	5'-GCCAGCATCACCGTATTATATTC-3'	
TXNRD2	hTR2RT-Fwd	5'-CAGCTTCAGGACAGAAAAGTC-3'	57
	hTR2RT-Rev	5'-GCTGACAGCAGAATCTCTTTC-3'	
GAPDH	GAPDHRT-Fwd	5'-GTGGTCTCCTCTGACTTCAAC-3'	55
	GAPDHRT-Rev	5'-GTCATACCAGGAAATGAGCTT-3'	
UPF1	Upf1RT-Fwd	5'-CAGGGCTACATCTCCATGAGC-3'	55
	Upf1RT-Rev	5'-CAAGGTAACTGTCCTGGGACAG-3'	

Table S3. Parameter values after fitting the models to the experimental data.

	Model 1			Model 2				Model 3			
mRNA	Transcription [1/s]	tRNAsec binding [ml/(mmol*s)]	NMD [1/s]	Transcription [1/s]	tRNAsec binding [ml/(mmol*s)]	NMD [1/s]	Ribosome drop-off [1/s]	Transcription [1/s]	tRNAsec binding [ml/(mmol*s)]	NMD [1/s]	Ribosome drop-off [1/s]
GPX1	1.59e-5	1.50e-4	6.09e-4	1.59e-5	1.50e-4	6.09e-4	1.64e-6	1.71e-3	7.74e-5	0.11	2.83e-3
GPX2	8.41e-6	1e-7**	1e-7**	8.41e-6	1e-7**	1.58e-7	60653	8.96e-4	9.73e-3	3.19	92.48
GPX4	2.38e-4	5.17e-5	9.56e-5	2.38e-4	5.20e-5	9.63e-5	2.59e-3	0.030	4.03e-6	4.91e-2	6.45e-6
SELH	2.38e-4	7.11e-5	3.33e-4	2.38e-4	7.15e-5	3.33e-4	2.65e-3	0.032	2.52e-5	0.091	2.87e-5
SELK	4.34e-5	1**	6.72e-6	/	/	/	/	/	/	/	/
SEP15	4.98e-6	1e-7**	1e-7**	/	/	/	/	/	/	/	/
SEPP1	6.15e-6	5.57e-7	7.89e-4	6.14e-6	6.83e-7	9.66e-4	0.097	2.00e-4	4.59e-7	3.66e-2	9.18e-5
SEPW1	9.36e-5	1.54e-4	5.29e-4	9.36e-5	1.57e-4	5.38e-4	0.035	1.93e-2	6.87e-5	0.17	4.34e-7
SPS2	2.17e-4	1e-7**	1e-7**	2.17e-4	1e-7**	1e-7**	93129	7.6e-3	1.35e-3	0.84	49.63
TXNRD1	8.35e-5	1.21e-7	9.02e-4	4.30e-4	1.21e-7	9.16e-3	0.020	9.67e-4	1.17e-7	1.88e-2	1.98e-3
TXNRD2	1.24e-4	3.72e-6	7.69e-5	1.24e-4	3.86e-6	7.98e-5	1.85e-3	3.44e-3	3.99e-7	1.14e-2	3.29e-3

** the parameter was not identifiable in the model with the available experimental data
